# Atypical or typical adrenocorticotropic hormone-producing pulmonary carcinoids and the usefulness of ^11^C-5-hydroxytryptophan positron emission tomography: two case reports

**DOI:** 10.1186/1752-1947-7-80

**Published:** 2013-03-19

**Authors:** Jeanette Wahlberg, Bertil Ekman

**Affiliations:** 1 Section of Endocrinology, Department of Medicine and Health Sciences, Faculty of Health, Sciences, Linköping University, Linköping, Sweden; 2 Department of Endocrinology, County Council of Östergötland, Linköping, Sweden

**Keywords:** ACTH, ACTH syndrome, Cortisol, Cushing’s syndrome, Ectopic, Pulmonary carcinoid

## Abstract

**Introduction:**

Pulmonary carcinoids associated with ectopic adrenocorticotropic hormone secretion have a good prognosis if histological examination shows typical pulmonary carcinoid and low proliferation, whereas a poor outcome is linked to atypical pulmonary carcinoid and high proliferation. Here we describe the diagnostic challenges to find the tumor in Cushing’s syndrome secondary to ectopic adrenocorticotropic hormone secretion in two cases with an atypical and a typical pulmonary carcinoid, respectively.

**Case presentation:**

A 63-year-old Caucasian woman presented with aggressive clinical features related to Cushing’s syndrome, having very high levels of urinary cortisol and circulating adrenocorticotropic hormone and cortisol. Magnetic resonance imaging showed no pituitary tumor, and bilateral inferior petrosal sinus sampling revealed no central peripheral ratio of adrenocorticotropic hormone. Computed tomography and ^111^Indium-pentetreoide somatostatin receptor scintigraphy could not visualize any ectopic tumor. The patient was referred for an ^11^C-5-hydroxytryptophan positron emission tomography, and a small 8mm nodule in her left lung was found. The tumor was removed via a lateral thoracic incision and wedge excision. The histological examination showed an atypical carcinoid with Ki-67 index of 9 to 10%, and an additional lobectomy was performed.

The second patient, a 22-year-old Caucasian man, also presented with aggressive Cushing’s syndrome, with very high urinary cortisol levels and increased circulating cortisol as well as adrenocorticotropic hormone levels. A magnetic resonance imaging scan of the pituitary showed no tumor, whereas a 12×9×14mm tumor was detected in the right lung on the primary computed tomography scan and no further investigation was performed. The tumor was removed via a lateral thoracic incision and wedge excision. A typical carcinoid with Ki-67 index of 1 to 2% was found and no further surgery was performed.

After surgical removal, the biochemical disturbances resolved and significant clinical improvement were achieved in both patients after 24 months of follow up.

**Conclusions:**

Diagnostic evaluation time is limited due to the aggressive course in ectopic adrenocorticotropic hormone-dependent Cushing’s syndrome. We suggest that ^11^C-5-hydroxytryptophan positron emission tomography could be considered early as a secondary diagnostic tool when primary computed tomography and/or magnetic resonance imaging scans fail to show any tumor.

## Introduction

Neuroendocrine tumors found in the thymus, thyroid, lungs, adrenals, gastrointestinal tracts and pancreas have been associated with ectopic adrenocorticotropic hormone (ACTH)- dependent Cushing’s syndrome (CS), with small cell lung carcinoma and pulmonary carcinoids accounting for the most cases [1-4]. In cases with a small cell carcinoma in the lung the radiological visualization of a tumor mass is in general obvious, whereas in ACTH-producing pulmonary carcinoids localization of the tumor could be unsuccessful using modalities like computed tomography (CT) and magnetic resonance imaging (MRI) of the patient's chest and abdomen [5]. Other techniques used to detect ectopic ACTH-secreting tumors with various results are somatostatin receptor scintigraphy (SRS) using ^111^indium-pentetreotide (^111^In-pentetreotide-SRS) [6] or technetium-99m-labeled octreotide acetate (Tc-99m-SRS) [7]. Pacak *et al*. found that fluorodeoxyglucose positron emission tomography (FDG-PET) did not detect ectopic ACTH-secreting tumors that were occult on CT and MRI [8], whereas ^11^C-5-hydroxytryptophan-PET (^11^C-5-HTP-PET) has been shown to visualize more and smaller lesions including pulmonary carcinoids compared with CT and SRS [5,9].

Here, we describe two cases with ACTH-producing pulmonary carcinoid tumor diagnosed preoperatively with ^11^C-5-HTP-PET and thoracic CT scan, respectively. The localization techniques and prognostic factors of pulmonary carcinoids are further discussed.

## Case presentation

### Case 1

A 63-year-old Caucasian woman was referred from a county hospital with an initial diagnosis of CS. She presented with generalized fatigue, muscle weakness, insomnia, flushing, hypertension, stress symptoms and psychiatric symptoms (deteriorated concentration, memory disabilities and hypomania). A physical examination revealed the typical clinical features of a severe CS: hypertension, changed body constitution, moon face, supraclavicular fat, buffalo hump, hirsutism on her chin, proximal myopathy, but also a weight loss (5kg over a few months). Her previous medical history included breast cancer (right side) 16 years earlier, cured with surgery and radiation. She had also had a superficial malignant melanoma on her right arm in year 2000 with no recurrence. Results of a biochemical test confirmed the diagnosis with 24-hour urine free cortisol (1300nmol/24-hour, reference <183). She had no diurnal rhythm and serum cortisol was around 600 to 1000nmol/L and plasma ACTH levels were between 30 and 45ng/L during the whole 24-hour sampling period. She did not suppress her cortisol and ACTH levels at all on either 1mg or 8mg dexamethasone suppression tests. An MRI scan of her sella turcica found no pituitary tumor. Bilateral inferior petrosal sinus sampling with corticotrophin-releasing hormone (CRH) testing revealed no central to peripheral ratio of ACTH, nor did ACTH increase during the CRH test. Fasting CRH was normal 3.5pmol/L (reference <5) and catecholamines and hydroxyindoleacetic acid in urine showed normal values. Chromogranin A was slightly elevated to 8.2nmol/L (reference <6). The patient did not have any proton-pump inhibitor. At this stage the clinical and imaging findings raised suspicion of an ectopic ACTH-producing tumor. A neck, thoracic and abdominal CT scan was done, showing only a slight enlargement of her left adrenal gland with attenuation of 25 Hounsfield units. Further, ^111^In-pentetreotide-SRS was performed in the whole body planar after an injection of 166 MBq ^111^Indium-labeled octreotide. The scan demonstrated no uptake typical for an ectopic ACTH source.

**Figure 1:**
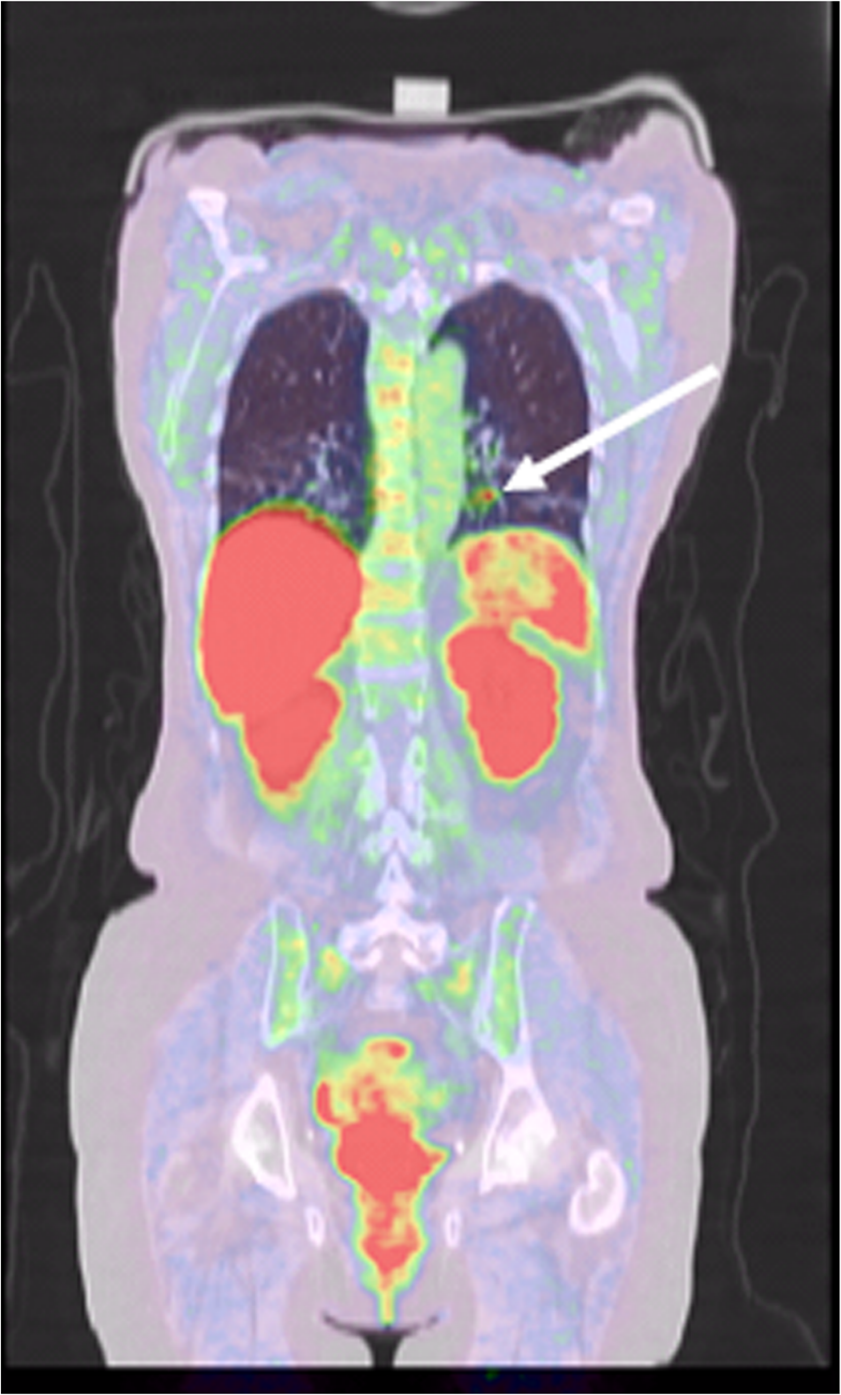
**Investigation with **^**11**^**C-5-hydroxytryptophan positron emission tomography shows an 8mm tumor in the left lower pulmonary lobe in a 63-year-old woman with aggressive ectopic adrenocorticotropic hormone-dependent Cushing’s syndrome.** Tumor indicated by an arrow.

Before we had the results from the sinus petrosus sampling, treatment with cabergoline was added, but it was quickly withdrawn due to side effects of dizziness, nausea, and uncontrollable shaking. The reason to start with cabergoline and not with ketoconazole was moderately elevated liver enzymes. However, despite the deterioration in liver enzymes the patient was commenced on ketoconazole 200mg twice daily. She also received metapyrone (metyrapone) in increasing doses up to 2.5g, which could not be increased more due to side effects with increased flushing and anxiety attacks. Regardless of the increase in dose of metapyrone (metyrapone) in addition to ketoconazole no clinical improvement was seen, and due to further increase in the deterioration in liver enzymes ketoconazole was discontinued. However, her cortisol and ACTH levels were stable and did not further increase during the investigational period. Due to the deterioration of her health condition a bilateral adrenalectomy was planned. Investigations with ^11^C-5 HTP-PET are centralized to one place in Sweden and finally we contacted the PET center in Uppsala, Sweden, and an ^11^C-5-HTP-PET was performed showing an 8mm mass in the left lung lower lobe with focal uptake (Figure 1). In retrospect, the same shadow was present on thoracic CT, previously assessed as a vessel. A thoracic surgeon was contacted to perform a wedge excision of the tumor via a lateral thoracic incision. Histology of the removed tissue showed an atypical carcinoid tumor with trabecular and focally insular pattern; a few areas were showing a marked degree of nuclear pleomorphism and hyperchromasia that is associated with the presence of mitotic figures (up to six per 10 high power fields, HPF). Immunohistochemistry stains were positive for neurone-specific enolase, protein gene product 9.5, chromogranin A, ACTH and synaptophysin. The hilar lymph nodes were benign. Three mitoses/10HPF and Ki-67 showed 9 to 10% of tumor cells in growing phase. The tumor was radically extirpated, unfortunately with a narrow margin.

After the surgery ACTH was below the detection limit (<5ng/L) with low morning cortisol levels below 100nmol/L, and replacement therapy with hydrocortisone was initiated.

Due to the finding of an atypical carcinoid and the narrow radical surgery an additional lobectomy was performed. The histology of the whole lobe showed no signs of recurrent tumor tissue and dissected regional lymph nodes were benign. A new ^11^C-5-HTP-PET was performed 6 and 24 months after the second operation without any sign of new endocrine tumors. A dual-energy X-ray absorptiometry (DXA) scan showed osteoporosis with T-scores of −3.0 both in L2 to L4 lumbar spine and in total hip and she was prescribed annual zoledronic acid infusions. At 24 months follow up, she still has low morning cortisol levels below 100nmol/L and plasma ACTH <5ng/L before hydrocortisone intake in the morning (total daily dose of 25mg).

### Case 2

**Figure 2:**
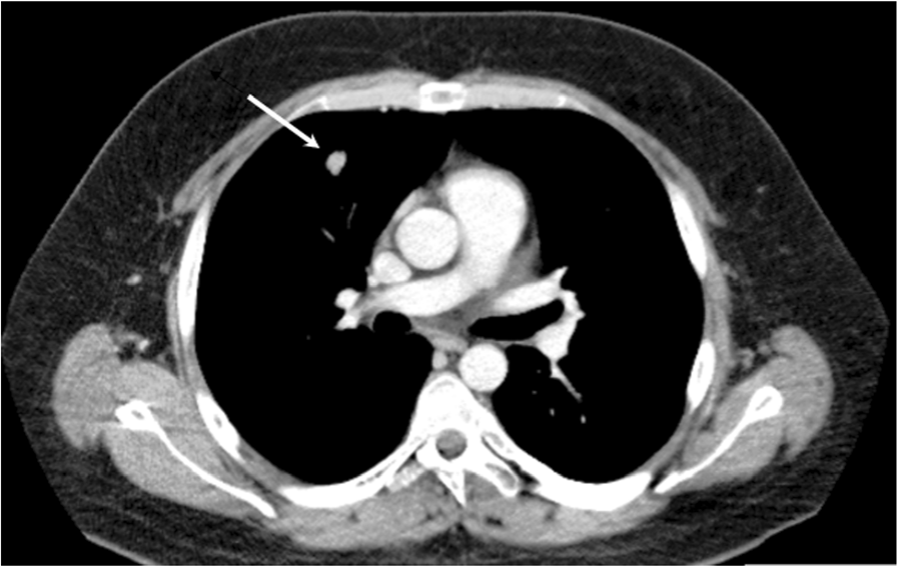
**Investigation with computed tomography shows a 12×9×14mm tumor in the right lower pulmonary lobe in a 22-year-old man with aggressive adrenocorticotropic hormone-dependent Cushing’s syndrome.** Tumor indicated by an arrow.

A 22-year-old Caucasian man presented with clinical features of a severe CS including centripetal fatness, moon face, severe edema, excessive purple striae on his trunk, and frequent nocturia. During the last years the patient had gained 30kg in weight despite diet and exercise plans. Despite a loss of 10kg in weight during the past months his centripetal fatness increased and he was so swollen that his eyes hardly could be seen. The serum cortisol concentration was 670nmol/L at 8:00 a.m., and 703nmol/L at 7.30 p.m. Plasma ACTH was 140ng/L at 8.00 a.m. (reference <46) and 140ng/L at 7.30 p.m. Sampling of 24-hour urinary free cortisol showed increased values, >3000nmol/24 hour (reference <183). He did not suppress his cortisol or ACTH levels at all on 1mg dexamethasone suppression testing. His chromogranin A levels and blood sugar levels were normal, whereas a very low serum testosterone of 2.6nmol/L (reference 7.6 to 31) was recorded. An MRI scan of his sella turcica did not visualize any pituitary tumor, whereas a pulmonary chest radiograph and subsequent CT scan revealed a 12×9×14mm diameter mass in the right lower pulmonary lobe (Figure 2). Because of the severe symptoms no further investigation was performed besides bronchoscopy and he was referred for subacute surgery. The tumor in the right lung was removed via a lateral thoracic incision by wedge excision. Histology confirmed a typical carcinoid with <1 mitosis/10HPF, and no necrosis was found. Ki-67 immunostaining was generally positive in 1 to 2% of the tumor cells and focally up to 5%. The tumor cells stained positive for ACTH, chromogranin A, and synaptophysin. Dexamethasone (1mg) suppression testing postoperatively showed suppression of cortisol to 46nmol/L, although ACTH suppressed only to 25ng/L. However, 24-hour urinary free cortisol was very low, revealing a value of 28nmol/24-hour. No further surgery was performed. Initially he achieved replacement therapy with hydrocortisone, but he stopped substitution after a few months. A DXA scan showed normal bone mineral density. At current follow up after 24 months he is in clinical remission and his diurnal urinary cortisol is still in the lower normal range.

## Discussion

Even if lung carcinoids have a better prognosis than other ACTH-secreting neuroendocrine tumors like small cell carcinomas, thymic carcinoids, medullary thyroid carcinomas and gastrinomas, they are a malignant disease with potential for metastases and increased mortality [1,10]. The present cases illustrate the dilemma between the need for morphological diagnosis of the ectopic ACTH source and control of the life-threatening hypercortisolism. A great help in our first patient was bilateral inferior petrosal sinus sampling with CRH stimulation for the differential diagnosis between pituitary and ectopic sources of ACTH [11], even if false positive cases have been reported [12]. There is no single diagnostic imaging technique for pulmonary carcinoid tumors. CT remains the gold standard for demonstrating abnormal mass in the chest [13]. In our second patient, a normal MRI scanning of the sellar region and a lung tumor identified with CT indicated ectopic origin of the ACTH-producing tumor, whereas in our first patient further investigation was needed.

The usefulness of Tc-99m-SRS has been described in selected cases with success [7] and may have advantages over ^111^In-pentetreotide-SRS used in our patient, with lower radiation dose and lower costs [6]. However, the tumor was not detected in our first patient and this could be due to possible lack of somatostatin receptors in the tumor or the size being only 8mm, which is near the resolution limit of 6mm for SRS methods [6]. FDG-PET was accessible, but this investigation did not visualize ectopic ACTH-secreting tumors that were occult [8]. Therefore we decided to refer the patient for ^11^C-5-HTP-PET instead. Neuroendocrine tumors have a capacity for uptake and decarboxylation of amine precursors like 5-HPT, which can be used as a tracer for PET imaging [5]. Compared with CT and SRS, ^11^C-5-HTP-PET has been shown to visualize more and smaller lesions down to 4 to 5mm [5,9]. Also, because of the biochemical pathway of 5-HPT, the nature of a lesion may be demonstrated (tumor, inflammation) [5,9] and, contrary to SRS, inflammatory lesions do not generally accumulate the tracer. In our first patient ^11^C-5-HTP-PET was essential for the diagnosis. The patient was only days from bilateral adrenalectomy, which is an effective treatment [14], but it would have had left her with a remaining atypical carcinoid in the lung with a poor prognosis [15]. We suggest that all modalities have to be used in searching for an occult ACTH-producing tumor, and in our first patient ^11^C-5-HTP-PET in an earlier stage had been preferable. Moreover, we did not perform any SRS or ^11^C-5-HTP-PET before primary surgery in the second patient as suggested by some authors [5], which has given us more uncertainty postoperatively regarding remaining tumor.

In our department the routine procedure for pulmonary carcinoids is surgery with wedge excision aiming to save lung parenchyma. Young age, central tumor, and no nodal enlargement are highly suggestive of typical carcinoid and recent guidelines advocate no further diagnostic or staging tests beyond chest CT and bronchoscopy before resection using parenchyma-sparing techniques [16]. However, some authors suggest that surgical removal of the lung carcinoid tumor should aim at “anatomic resection” rather than “parenchyma saving” surgery. This can be true in older patients and women who more often have small nodules with diameters of less than 5mm that are histologically similar to carcinoids (tumorlets) [16]. Search for involved mediastinal lymph nodes should be rigorous, and a low threshold for clearing lymph nodes should be adopted also in typical carcinoids [17]. Failure to remove all involved lymph nodes could lead to persistence or recurrence of the syndrome, and diminishes the chances of long-term survival [15]. Serum ACTH could be used as a tumor marker to predict early recurrence. Besides ACTH, these tumors were shown to secrete a number of biologically active hormones and precursors that could cause Cushing’s syndrome. CRH, corticotrophin-like intermediate lobe peptide, ACTH precursors, and pro-opiomelanocortin were among the described factors [18].

Taken together, if the tumor can be visualized with SRS or ^11^C-5-HTP-PET preoperatively in addition to laboratory specimens the post-surgery follow up is facilitated [5].

## Conclusions

Diagnostic evaluation time is limited due to the aggressive course in ectopic ACTH-dependent CS. We suggest that a referral to a specialized center for investigation with ^11^C-5-HTP-PET in selected cases is preferable when a CT and MRI examination fail to detect any ectopic ACTH-producing tumor.

## Consent

Written informed consent was obtained from the patients for publication of this case report and any accompanying images. A copy of the written consents are available for review by the Editor-in-Chief of this journal.

## Competing interests

The authors declare that they have no competing interests.

## Authors’ contributions

JW and BE both participated in the design and coordination of the case reports, drafting the manuscript and interpreting the radiological figures and revised the article for important intellectual content and helped draft the manuscript. Both authors read and approved the final manuscript.
